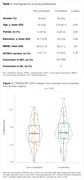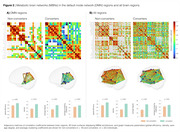# Metabolic PET brain networks predict clinical conversion prior to amyloid positivity in cognitively unimpaired individuals

**DOI:** 10.1002/alz.094094

**Published:** 2025-01-09

**Authors:** Christian Limberger, Gabriel Colissi Martins, Giovanna Carello‐Collar, Thomas Hugentobler Schlickmann, Guilherme G. Schu Peixoto, Marco Antônio de Bastiani, Luiza Santos Machado, Guilherme Povala, Tharick A. Pascoal, Pedro Rosa‐Neto, Débora Guerini de Souza, Eduardo R. Zimmer

**Affiliations:** ^1^ Universidade Federal do Rio Grande do Sul, Porto Alegre, Rio Grande do Sul Brazil; ^2^ Universidade Federal do Rio Grande do Sul, Porto Alegre, RS Brazil; ^3^ University of Pittsburgh, Pittsburgh, PA USA; ^4^ McGill Centre for Studies in Aging, Montreal, QC Canada; ^5^ Universidade Federal do Rio Grande do Sul, Porto Alegre Brazil

## Abstract

**Background:**

The default‐mode network (DMN) consists of brain regions with higher resting activity levels. Amyloid‐ß (Aß) deposition in Alzheimer’s disease (AD) occurs predominantly throughout the DMN, suggesting that activity within the network may facilitate disease processes. Indeed, increased neural activity is positively associated with Aß production. In this context, variations in DMN activity and associated metabolic networks may be linked to the risk of developing AD. However, how patterns of metabolic disruption relate to the progression of AD pathology remains unknown. Here, we investigated whether the metabolic brain networks (MBNs) architecture predicts clinical conversion in cognitively unimpaired (CU) individuals.

**Method:**

We selected CU individuals negative to amyloid and tau (A‐T‐) from the ADNI cohort with [18F]FDG‐PET imaging data at baseline. These patients were divided in stable (non‐converters, n = 18) and clinical progressors (converters, n = 22). Individuals were age‐ and APOEe4‐matched (Table 1). The mean [18F]FDG standard uptake value ratio (SUVR, pons as reference) of brain regions of interest (ROIs) was extracted based on the DKT atlas. MBNs were assembled with a multiple sampling bootstrap scheme and corrected for group imbalance with the Adaptive Synthetic Sampling Approach for Imbalance (ADASYN) and for multiple comparisons using FDR (p < 0.05).

**Result:**

[18F]FDG regional SUVRs presented no differences between groups (Figure 1). However, converters had a prominent brain PET metabolic hyperconnectivity compared to non‐converters, with a 1.5 fold‐change in connection density (p < 0.001, Figure 2A). Notably, this hyperactivation was not limited to the ROIs comprising the DMN; MBNs constructed with all brain regions reveal that the brains of converters typically display metabolic hyperactivity before the onset of CI (Figure 2B).

**Conclusion:**

Our findings suggest the existence of early metabolic alterations at the network level in amyloid negative converters. This corroborates the notion that early soluble forms of amyloid, considered synaptoxins, may trigger brain metabolic hyperconnectivity. MBNs hold promise as biomarkers for detecting individuals at risk of clinical progression, even before amyloid positivity status.